# Minimum viable population size and population growth rate of freshwater fishes and their relationships with life history traits

**DOI:** 10.1038/s41598-019-40340-z

**Published:** 2019-03-05

**Authors:** Teng Wang, Masami Fujiwara, Xin Gao, Huanzhang Liu

**Affiliations:** 1 Key Laboratory of Aquatic Biodiversity and Conservation of Chinese Academy of Sciences, Institute of Hydrobiology, Chinese Academy of Sciences, Wuhan, Hubei China; 2 Key Laboratory of South China Sea Fishery Resources Exploitation & Utilization, Ministry of Agriculture, South China Sea Fisheries Research Institute, Chinese Academy of Fishery Sciences, Guangzhou, Guangdong China; 3 Department of Wildlife and Fisheries Sciences, Texas A&M University, College Station, TX 77843-2258 USA

## Abstract

The minimum viable population (MVP) size has been compared for a wide range of organisms in conservation biology, but a limited number of studies investigated it for freshwater fishes, which exhibit diverse life history strategies. In this study, the MVP size and population growth rate of 36 fish species in the Yangtze River were estimated and compared with their life-history traits. The results indicated that the MVP size ranged from 42 to 320 individuals, and instantaneous per-capita population growth rate ranged from 0.009 to 0.188 per year. MVP size and population growth rate were significantly associated with three life history traits: the age at maturity, generation time, and fecundity. Long-lived species with delayed maturation, long generation time, and high fecundity had a greater MVP size and a lower population growth rate than short-lived species. Therefore, our results emphasize a need for prioritizing our conservation effort more on long-lived species.

## Introduction

Freshwater fishes are confronting some of the most serious threats in the world, including habitat loss and fragmentation, pollution, species invasion, and overfishing^1^, which caused freshwater fishes to experience a high risk of extinction^2^. Conservative estimates of population viability suggested that 1800 species, which accounted for 20% of all freshwater fishes, had become extinct or on the verge of extinction in 1992^2^. To make the situation worse, the number of endangered freshwater species on the International Union for the Conservation of Nature (IUCN) Red List has more than tripled since 2003^3^. Although this is partly because we have more data now than 2003, permitting us to categorize them as threatened, these facts are prompting need for a comprehensive effort to assess the viability of freshwater fishes.

Two of the important measures of the viability of species are minimum viable population (MVP) size and annual population growth rate. MVP size is the smallest size in which a population can persist despite of confronting impacts from various factors^4–8^. In practice, MVP is usually estimated to be equal to the population size enough to ensure a pre-specified probability of persistence over a pre-specified period into the future^1,9^. MVP has become particularly useful to guide conservation efforts for species at risk by providing science-based target abundance in a recovery plan^10,11^. For example, IUCN Criterion D categorizes the level of threat based on a set of threshold population sizes, whilst Criterion E uses the projected risk of extinction faced by a species over a specified period (IUCN 2005). Traill *et al*.^12^ indicated the number of studies using MVP has gradually increased over a past few decades with no apparent sign for decline in its use. This is partly because conservation agencies need to make rapid decisions about conservation targets, often under limited data availability. At the same time, it is also attracting interests in developing a robust, general guideline for determining MVPs^13,14^. Such a guideline could utilize basic life history traits or other ecological attributes^13,15^, which are more readily available. To develop the guideline, the analyses of MVP using multiple species have been performed^12,13^. Nevertheless, these studies, which depended on multiple data types from multiple species, could not derive such a guideline conclusively. Furthermore, for some taxa such as fish, data are lacking to conduct simultaneous population viability analyses of multiple species^16^, leading to a limited number of comprehensive research on the MVP of fish.

Population growth rate is another measure often used in viability analysis. It is a per-capita rate at which the number of individuals in a population increases/decreases over a fixed period. Hutchings^17^ argued that population growth rate ultimately determined fish population’s ability to sustain under fishing mortality and to recover after its collapse. Therefore, population growth rate could be used to assess the intrinsic vulnerability and extinction risk of fish species^18,19^, and has been adopted as one of the most important indices by the IUCN Red List guideline and many national guidelines and criteria (e.g. American Fisheries Society)^20^. However, in practice, population growth rate is very difficult to estimate, especially, for endangered species due to a lack of demographic data^19^. Instead, the correlations between life-history traits and population growth rate have been quantified in order to evaluate vulnerability of species under threats from various environmental and anthropogenic factors^19,20^.

The objectives of this study were to (1) calculate the MVP and population growth rate of fishes in the Yangtze River, (2) estimate the relationships between the two population viability measures and life-history traits, and (3) suggest conservation strategies for fishes in the Yangtze River.

## Results

### Minimum viable population size and population growth rate

Our results indicated that the minimum viable population sizes of 36 fish species ranged from 42 to 320 individuals with the average value of 117 (SD: 66) individuals (Table 1). *Pseudobrama simoni* and *Hypophthalmichthys nobilis* had the maximum and minimum values of MVP, respectively. The MVP values for *Mylopharyngodon piceus*, *Ctenopharyngodon idellus*, *Schizopygopsis malacanthus chengi*, *Xenocypris microlepis* were more than 200 individuals. There were 17 species whose MVP values were less than 100 individuals. The MVP of 6 species, *Pseudobrama simoni*, *Xenophysogobio boulengeri*, *Botia superciliaris*, *Glyptothorax fukiensis*, *Channa argus*, and *Culter alburnus*, were less than 60 individuals.

**Table 1:** Minimum viable population sizes (MVPs) and population growth rate (*r*) of thirty-six fish resulted from the outputs of VORTEX model.

Species	Minimum viable population sizes (MVPs)	Population growth rate (*r*)
*Odontobutis obscura*	80	0.155
*Pseudobrama simoni*	42	0.188
*Ancherythroculter kurematsui*	92	0.137
*Pelteobagrus fulvidraco*	66	0.145
*Sarcocheilichthys nigripinnis*	68	0.135
*Xenophysogobio boulengeri*	50	0.181
*Botia superciliaris*	56	0.116
*Glyptothorax fukiensis*	56	0.182
*Ancherythroculter nigrocauda*	104	0.115
*Pseudogobio vaillanti*	136	0.076
*Paracanthobrama guichenoti*	118	0.076
*Pseudobagrus truncatus*	84	0.099
*Pelteobagrus vachelli*	110	0.089
*Sinibrama macrops*	72	0.1
*Squalidus argentatus*	110	0.1
*Coreius heterodon*	62	0.113
*Misgurnus anguillicaudatus*	64	0.13
*Cultrichthys erythropterus*	110	0.107
*Leptobotia rubrilabris*	110	0.102
*Channa argus*	58	0.115
*Megalobrama pellegrini*	70	0.115
*Xenocypris microlepis*	214	0.06
*Culter alburnus*	54	0.122
*Rhinogobio cylindricus*	68	0.117
*Hypophthalmichthys molitrix*	136	0.082
*Saurogobio dabryi*	148	0.052
*Garra pingi*	80	0.08
*Cyprinus* (*Cyprinus*) *carpio*	198	0.054
*Paracanthobrama guichenoti*	114	0.055
*Schizopygopsis malacanthus chengi*	220	0.015
*Ctenopharyngodon idellus*	230	0.039
*Hypophthalmichthys nobilis*	320	0.027
*Schizothorax sinensis*	180	0.045
*Silurus meriordinalis*	120	0.04
*Mylopharyngodon piceus*	240	0.024
*Acipenser sinensis*	180	0.009

The present results indicated that the instantaneous population growth rate (*r*) ranged from 0.009 to 0.188 with the average of 0.094 (SD: 0.047) (Table 1). *Pseudobrama simoni* and *Acipenser sinensis* had the maximum and minimum values of the population growth rate, respectively. The population growth rates of 17 species were below 0.1; these species included *Acipenser sinensis*, *Schizopygopsis malacanthus chengi*, *Mylopharyngodon piceus*, and *Hypophthalmichthys nobilis* (Table 1). The population growth rates of 19 species were more than 0.1; these species included *Pseudobrama simoni*, *Glyptothorax fukiensis*, *Xenophysogobio boulengeri* (Table 1).

### Relationships between two population viability measures and life-history traits

**Figure 1:**
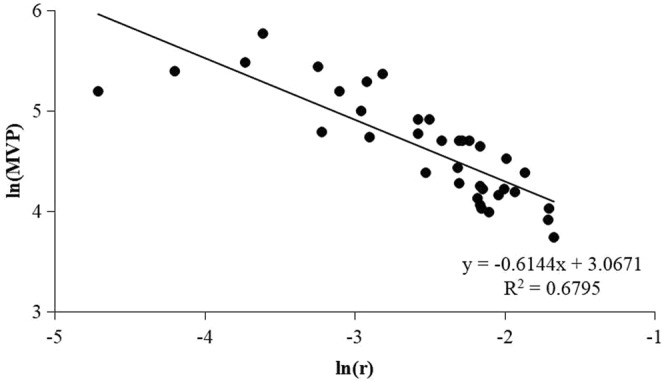
Relationships between natural log-transformed minimum viable population (MVP) and population growth rate (*r*) of 36 fish species.

**Figure 2:**
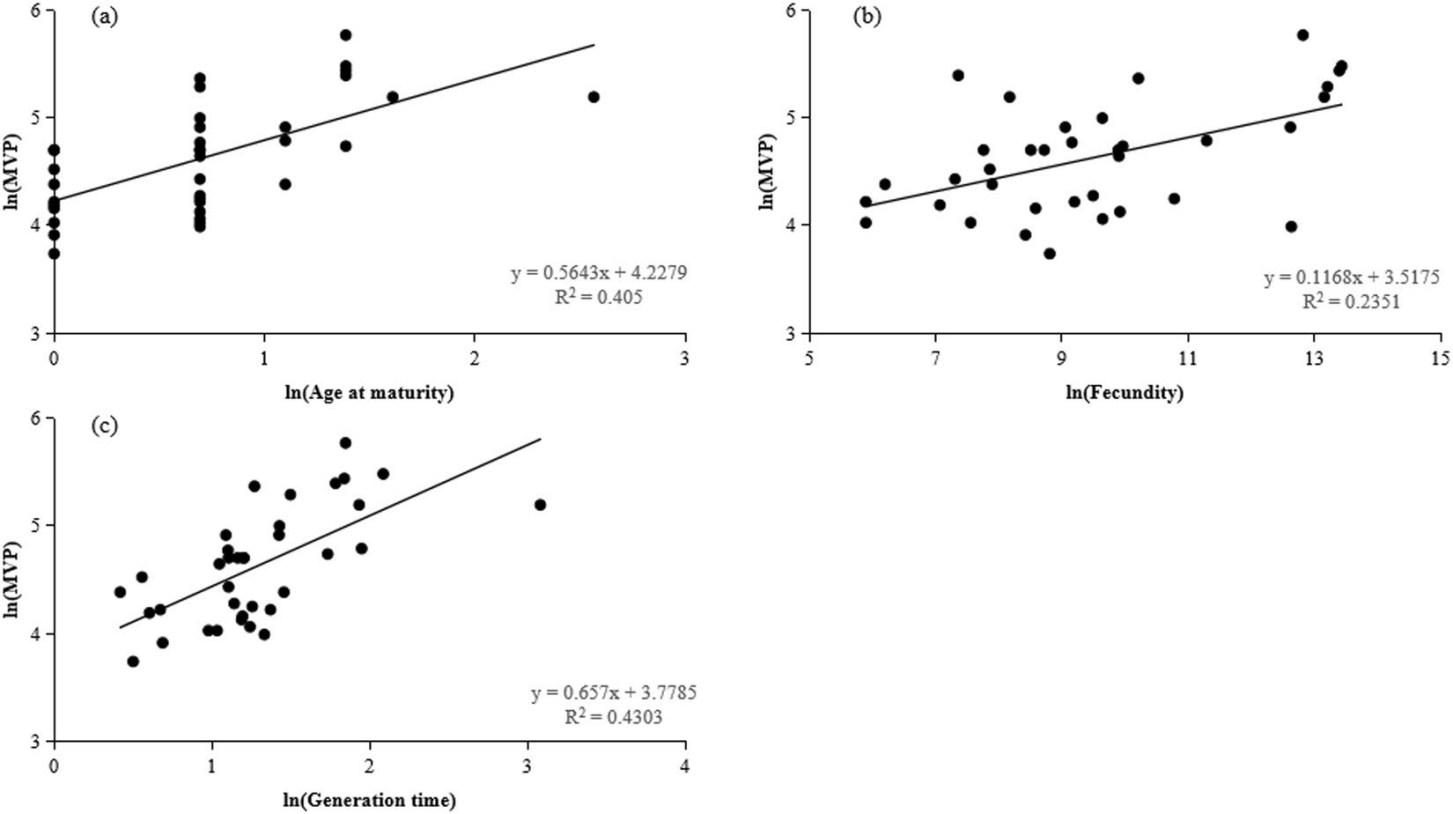
Relationships between natural log-transformed minimum viable population (MVP) sizes and life history traits of 36 fish species, including age at maturity (**a**), fecundity (**b**), and generation time (**c**).

**Figure 3:**
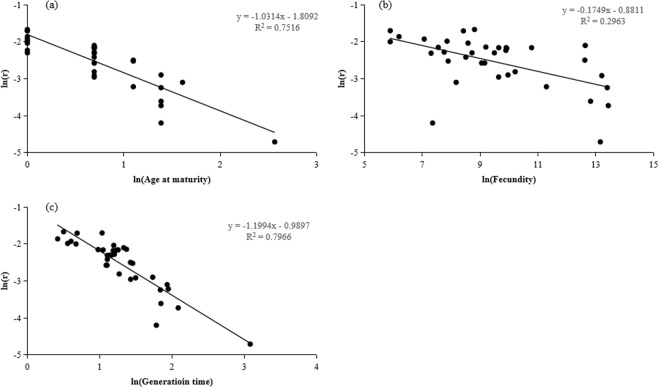
Relationships between natural log-transformed population growth rate (*r*) and life history traits of 36 fish species, including age at maturity (**a**), fecundity (**b**), and generation time (**c**).

There was a significantly negative correlation between the natural log-transformed MVP and *r* (*R*^2^ = 0.6795, *P* < 0.001) (Fig. 1). Linear regression indicated that the natural log-transformed MVP sizes were significantly and positively related to the natural log-transformed age at maturity (*R*^2^ = 0.405, *P* < 0.001) (Fig. 2a), fecundity (*R*^2^ = 0.2351, *P* < 0.05) (Fig. 2b), and generation time (*R*^2^ = 0.4303, *P* < 0.001) (Fig. 2c). Linear regression also showed that the natural log-transformed population growth rates (*r*) were significantly and negatively related to the natural log-transformed age at maturity (*R*^2^ = 0.7516, *P* < 0.001) (Fig. 3a), fecundity (*R*^2^ = 0.2963, *P* < 0.001) (Fig. 3b), and generation time (*R*^2^ = 0.7966, *P* < 0.001) (Fig. 3c).

## Discussion

### Minimum viable population size

Franklin^21^ has proposed the famous 50/500 rule for minimum effective population size, which has become the threshold to prevent inbreeding depression^22^. This rule specifies that the genetic effective population size (*N*_*e*_) should not be less than 50 in a short term and 500 in a long term. MVP size (*N*_*c*_) is the threshold above which a population can persist over a specified period with a specified probability considering the demography of the population. Although *N*_*e*_ and *N*_*c*_ are different, the 50/500 rule is generally used to provide guidance for assessing MVP in the absence of demographic data for many endangered species^12,23,24^. Frankham^25^ comprehensively estimated the average ratio between effective population size to MVP (*N*_*e*_/*N*_*c*_) of 102 species to be 0.10–0.11. Rosenfeld^26^ suggested that the MVP should be from 5 to 10 times the effective population size. Then, the MVP should be between 250 to 2500 in a short term and 2500 to 5000 in a long term.

Based on the review of the literature, it has been suggested that MVP size in general should be in the hundreds^9^. However, much greater guideline level for MVP has also been suggested^26^. For example, Reed *et al*.^23^ estimated the mean and median values of MVP of 102 vertebrate populations were 7316 and 5816 adults, respectively. Reed^27^ also suggested that a population should have more than 2000 individuals in order to maintain a healthy population for its long-term persistence. Brook *et al*.^13^ estimated MVP for 1198 species, and indicated the median MVP estimate was 1377 individuals. Traill *et al*.^12^ revealed that a median value of MVP of 212 species was 4169 individuals based on 30 years of published estimates. Traill *et al*.^24^ indicated that there should be at least 5000 adults for a species persistence. These variations probably reflect both uncertainty in MVP due to multiple factors affecting it and variation in the choice of the probability and duration of persistence used in the studies (Table S1).

Our results indicated that the MVP sizes of 36 fish species in the Yangtze River were much less than the aforementioned guideline values. The previously proposed high MVP values probably resulted from four reasons. First, few r-selection species, common among many fish species, were included in the previous studies^12^. For example, Vélez-Espino and Koops^11^ indicated that the average MVP value of 31 freshwater fishes in Canada was 272 adults (range: 51–1350) without impacts of the catastrophes. Second, the effects of inbreeding depression were assumed very strong on the natural populations^27,28^. Third, the effects of catastrophic events have been overestimated^29^. Finally, we selected a higher end of value for the probability of persistence.

Catastrophic events are defined as low-frequency high-amplitude events^30^. As they are high amplitude events, they affect MVP^11^. In our analysis, we did not incorporate catastrophic events; consequently, MVP is underestimated if catastrophic events were to happen. However, we did not feel it is appropriate to include an arbitrary reduction in a vital rate with an arbitrary frequency as an effect of catastrophic events. We suggest it is important to make the prediction of the spatial extent of catastrophic events and compare it with the spatial distribution (or the spatial extent of essential habitat) of the species of interest. Then, we can incorporate the inverse of the proportion of species unaffected by the catastrophic events as a multiplier on the MVP to adjust the value. However, in the current analysis, we did not have neither prediction of the catastrophic events nor the information of the species distribution for a large number of species. Therefore, our MVP should be treated as the base-line values that can be adjusted for catastrophic events when such information become available.

The single value MVP threshold approach may not apply to all species in general because there are also many studies that suggested different and lower MVP for specific species. For example, the MVP of grizzly bears (*Ursos arctus horribilis*) was 125^31^; Japanese black bears (*Ursus thibetanus japonicus*) was 100^32^; wild boar (*Sus scrofa*) was 300^33^; Asian elephants (*Elephas maximus*) was approximately 120^34^; bog turtle (*Glyptemys muhlenbergii*) without inbreeding was 15^35^; medicinal leech (*Hirudo medicinalis*) was 248^36^; and lake sturgeon (*Acipenser fulvescens*) with inbreeding effects was 150^37^. Shoemaker *et al*.^35^ researched the MVP of long-lived organisms and concluded currently suggested MVP thresholds^12,23^ may be 1–2 orders of magnitude too high even for long-lived organisms. Our results are more consistent with the results from these studies.

Moreover, other anecdotal evidence suggested lower MVP. For example, some of the island bird populations studied by Jones and Diamond^38^ may have survived for 80 years despite possessing median numbers of fewer than 10 pairs. Another example is northern elephant seals, which recovered from about 20 to at least 30,000 individuals over a period of 75 years^39^. Berger^40^ evaluated the persistence of isolated populations of bighorn sheep (*Ovis canadensis*). Populations <50 individuals went locally extinct in less than 50 years, while those containing ≥100 individuals generally persisted up to 70 years. Since these are probably only a few of many populations experienced a low population abundance (i.e. there are also many that actually went to extinct after experiencing low population abundance), these are merely anecdotal evidence. Nevertheless, the contradictions in the existing literature with regard to MVP suggested that a one-fits-all approach is not appropriate and that further studies are needed.

### Relationships between minimum viable population and life-history traits

The present results indicated that the MVP was significantly associated with the life history traits. This is consistent with the result of Harcourt^41^, who suggested the MVP of the large-body species is more than that of the small-body species. Because large body is often associated with long life^42^, we expected to have the association between life history traits and MVP. On the other hand, our results contrasts with Brook *et al*.^13^, who suggested that MVP size was more strongly related to local environmental variations than life history traits. However, they included 1198 species belonging to a wide range of taxa, which probably obscured existing patterns, whereas our study focused on fishes in the Yangtze River. Our results are also consistent with a theoretical study by Fujiwara^43^, who demonstrated differences in extinction threshold among fishes of similar taxa incorporating demographic stochasticity alone. Therefore, we suggest our results could be used for developing a quick conservation guideline for organisms within similar taxa such as freshwater fishes using their life history traits.

Our result showed long-lived fishes (older age of maturation and longer generation time) had higher MVP sizes, suggesting we must maintain them at high population size compared with short-lived organisms. This complicates conservation effort because long-lived organisms tend to be suppressed in its abundance substantially relative to its carrying capacity to achieve the maximum sustainable yield (MSY)^44^. In other words, the difference between population sizes at MVP and MSY are expected to be small for long-lived fishes.

### Relationships between population growth rate and life history traits

The present results indicated that the population growth rate was significantly negatively correlated with some life history traits, such as age at maturity, generation time, and fecundity (Fig. 2). Asymptotically, all populations should have an instantaneous population growth rate of 0 regardless of life history strategies if they were to persist. However, the estimated population growth rate reflects the status of the fish populations when data were collected. The result suggests longer-lived fishes were affected more severely by the conditions they experienced.

Our result on population growth rate was consistent with some of the previous studies. Morato *et al*.^45^ indicated that the biological characteristics, such as longer life, later maturation, and slower growth, were subject to greater vulnerability. Jennings *et al*.^46^ found that larger and later maturing species were more vulnerable to fishing pressure than smaller and earlier maturing species. Heppell *et al*.^42^ also showed that the extinct and endangered marine animals were characterized by relative large body and long life.

### Conservation strategies for fishes in the Yangtze River

The present results demonstrated that the longer-lived fishes with late maturity, long generation, and large fecundity were expected to have a greater MVP size than the shorter-lived fishes in the Yangtze River. This implies that in spite of playing an important role in the structure and function of an ecological community, long-lived species tend to be at a relative high risk of extinction and are vulnerable to natural and anthropogenic disturbances^47–49^. Hence, we should place priority on long-lived species for conservation effort in the Yangtze River.

Many factors, such as hunting/fishing, habitat alteration, invasive species, and climate change, could cause the population decline of long-lived species, which also tend to be less resilient and slow to adapt to changing environmental conditions^42,50–55^. Some problems such as climate change is very difficult to solve in a short term^56^, and some alterations of habitat, such as construction of dams, are practically almost impossible to restore to historical conditions. On the other hand, other types of conservation efforts such as reduction in fishing pressure and creation of reserves can be implemented quickly. The previous studies found that increasing the survival of both the juveniles and adults was most beneficial to the persistence of long-lived species^57–60^. Furthermore, stock enhancement can potentially be implemented to mitigate habitat alternations. Our results suggest these conservation efforts should be allocated more toward protecting long-lived species in the Yangtze River.

## Methods

### Ethics statement

All methods used in this study were conducted in accordance with the Laboratory Animal Management Principles of China. All experimental protocols in this study were approved by the Ethics Committee for Animal Experiments of the Institute of Hydrobiology, Chinese Academy of Sciences.

### Minimum viable population

In the present study, minimum viable population (MVP) was defined as the minimum number of individuals that was sufficient to sustain 99% probability of population persistence in 100 years^5^. Moreover, we incorporated a genetic effect into the estimate of MVP by considering viable population to maintain more than 90% of genetic diversity, as proposed by Frankham *et al*.^61^. We estimated their MVP sizes and population growth rates using VORTEX version 10.0.7.3^62^. VORTEX, which is an individual-based population simulation model, performs Monte Carlo simulations incorporating the effects of deterministic forces, such as habitat loss and over-exploitation, as well as demographic, environmental, and genetic stochasticity^62,63^. The software has been extensively used to assess the extinction risk of endangered species by the Conservation Breeding Specialist Group of IUCN and other conservation and land management agencies^64,65^.

### Estimating parameters

Biological and demographic data of 36 fish species, belonging to 8 families and 4 orders, in the Yangtze River were collected for estimating the parameters to be inputted into VORTEX (Table S2). The population model of each species was run over 100 years for 1000 iterations. Population growth rate and extinction probability were provided as the outputs. The time step was set to 365 days, namely one year. A population was considered extinct when only individuals in at most one sex remained. Environmental variation (EV) was set at 10% as suggested by Medici and Desbiez^66^. To investigate the sensitivity of the results to the magnitude of EV, we also increased it to 20% and 30%. However, the estimated MVPs were affected only slightly (Table S3), and the qualitative results (ranking of the results among species) did not change. The percentage of adults (both females and males) reproducing each year was set at 100% for all species because all fishes, except Chinese sturgeon, exhibit annual reproduction. Adult Chinese sturgeon spawn once every two years^60^. To accommodate it, we reduced the annual fecundity by 50%. Sex ratio of offspring was assumed as 1:1. The age of first breeding, which is in accordance with the age of maturity, and maximum lifespan were sourced from Wang *et al*.^60^.

For age *t* ≥ 1 year, mortality rate was estimated by combining the three methods of Pauly^67^, Jensen^68^, and Chen and Watanabe^69^ for each species separately. The three methods are as following:

Pauly^67^:

1 $$\mathrm{ln}\,(M)=-\,0.0152-0.279\,\mathrm{ln}\,({L}_{\infty })+0.6543\,\mathrm{ln}\,(k)+0.463\,\mathrm{ln}\,(T){\rm{.}}$$

Jensen^68^:

2 $$M=1.6\,k.$$

Chen and Watanabe^69^:

3 $${M}_{(t)}=\{\begin{array}{cc}\frac{k}{1-{e}^{-k(t-{t}_{0})}}, & t\le {t}_{mat}\end{array}$$

4 $${M}_{(t)}=\{\begin{array}{cc}[\frac{1}{{t}_{{\rm{\max }}}-t}]\times \,\mathrm{ln}[\frac{{e}^{k}{t}_{{\rm{\max }}}-{e}^{k}{t}_{0}}{{e}^{k}t-{e}^{k}{t}_{0}}], & t\ge {t}_{mat}\end{array}$$

where *L*_*∞*_ is the asymptotic length, *t*_max_ is the maximum age in a population (life span), *k* is the growth coefficient, *t*_0_ is the age at length 0, *t*_*mat*_ is the age at maturity (Table S2), *T* is the annual mean water temperature (18 °C)^70^. For age *t* ≥ *t*_*mat*_, the mortality rate at each age class was set at the mean of the mortality rates calculated with Eqs (1, 2 and 4). For age 1 ≤ *t* < *t*_*mat*_, the mortality rate at each age class was approximated with Eq. (3).

The instantaneous natural mortality rate from egg to age 1 (*M*_0_) and fecundity (*F*) were obtained from Wang *et al*.^60^ (Table S2). However, the fecundity was so large that the model simulation was not feasible. Hence, according to VORTEX user’s manual^62^, we first calculated the number of age 1 produced per female by multiplying *F* and ${e}^{-{M}_{0}}$ (Table S2), and then, we back-calculated per-capita fertility rate *B*, which gives the number of age 0 produced per female, assuming that 10% of them survived from age 0 to age 1 as

$$B=\frac{{e}^{(-{M}_{0})}\times F}{1-0.1}.$$

This way of calculating the fertility rate *B* reduced the number of offspring produced, allowing the simulations, but it maintained the expected number of age 1 according to the estimated fecundity and survival rate.

We used the Poisson distribution for the number of offspring per female per brood^62^. The Poisson distribution was simulated for 1000 times in order to get a distribution^23^.

We evaluated the effect of carrying capacity on estimated MVP, population growth rate, and genetic diversity by modeling the populations of *Pseudobrama simoni* and *Acipenser sinensis* with a range of carrying capacity at 500, 1000, 2000, 5000, and 10000. The results indicated that there was no significant difference in neither population growth rate nor minimum viable population size among different carrying capacity levels (Table S4). This is because the model assumes constant mean per capita rates (i.e. density independence) up to the carrying capacity, and what happens to a population at high population abundance near a carrying capacity should not affect the viability of population significantly unless the carrying capacity is set very low. However, the genetic diversity of *Pseudobrama simoni* was lower than 90% with the carrying capacity of 500. Based on these preliminary results, carrying capacity was set at 1000 for estimating MVP and 5000 for estimating population growth rate. The latter value was set higher to allow population to grow for a sufficiently long period of time before reaching the carrying capacity.

Effect of inbreeding depression was incorporated into a model with the default value of 6.29 for lethal equivalents to estimate MVP sizes due to the small population, but it was not incorporated for estimating population growth rate. The initial population size to estimate population growth rate was set at 500 individuals, which is the maximum value of 50/500 rule proposed by Franklin^21^. In the outputs, the stochastic instantaneous population growth rate (*r*) was given by the mean rate of stochastic instantaneous population growth or decline exhibited by the simulated populations, and it was obtained by averaging across years and iterations for all simulated populations that are not extinct.

### Relationships between two population viability measures and life-history traits

The three parameters, age at maturity, fecundity, and generation time, were used to analyze the relationships between the life-history traits and the two population viability measures (MVP and *r*). All of the life history traits and two population measures were transformed by taking natural logarithm to stabilize variance. At first, the relationship between the natural log-transformed MVP and *r* was analyzed, and then, the relationships between the natural log-transformed life history traits and two population viability measures were analyzed with linear regression.

## Supplementary information

Supplementary Information

## Data Availability

The datasets generated during and/or analyzed during the current study are available from the corresponding author on reasonable request.
