# Supplementary material for: Minimum viable population size and population growth rate of freshwater fishes and their relationships with life history traits
Source: Sci Rep. 2019 Mar 5;9:3612. doi: 10.1038/s41598-019-40340-z (PMC6401379; doi:10.1038/s41598-019-40340-z)
Supplement: Supplementary file 1 — Supplementary Information [file 41598_2019_40340_MOESM1_ESM.pdf]

Supplementary Information

Minimum viable population size and population growth rate of freshwater fishes and their relationships with life history traits

Teng Wang[1, 2], Masami Fujiwara[3], Xin Gao[1, *], Huanzhang Liu[1, *]

[1] Key Laboratory of Aquatic Biodiversity and Conservation of Chinese Academy of Sciences, Institute of Hydrobiology, Chinese Academy of Sciences, Wuhan, Hubei, China

[2] Key Laboratory of South China Sea Fishery Resources Exploitation & Utilization, Ministry of Agriculture, South China Sea Fisheries Research Institute, Chinese Academy of Fishery Sciences, Guangzhou, Guangdong, China

[3] Department of Wildlife and Fisheries Sciences, Texas A&M University, College Station, TX, 77843-2258, USA

[*] Corresponding. hzliu@ihb.ac.cn, gaoxin@ihb.ac.cn

Table S1 Minimum viable population (MVP) sizes. MVP's were estimated with the different probability and duration of persistence in the previous studies

References	Species	Minimum viable population (MVP)	Probability of persistence	Time of persistence
Reed et al. [23]	102 vertebrate	7316 and 5816 adults	99%	40 generations
Reed [27]	11 species	2000	≥80%	≥20 years
Brook et al. [13]	1198 species	1377	90%	100 years
Traill et al. [12]	212 species	4169	80-100%	3-1200 generations
Traill et al. [24]	-	5000	-	-
Vélez-Espino and Koops [11]	31 species	272 adults (range: 51-1350) without impacts of the catastrophes	95%	250 years
Suchy et al. [31]	Grizzly bears	125	95%	100 years
Horino & Miura [32]	Japanese black bears	100	95%	100 years
Howells & Edwards-Jones [33]	Wild boar	300	95%	50 years
Armbruster et al. [34]	Asian elephants	120	>90% (93-96%)	1000 years

Shoemaker et al. [35]	Bog turtle	15 breeding females	>90%	100 years
Elliott et al. [36]	Medicinal leech	248	-	-
Schueller & Hayes [37]	Lake sturgeon	150	95%	250 years
Jones & Diamond [38]	Island bird	< 10 pairs	-	80 years
Bonnell & Selander [39]	Northern elephant seal	20	-	75 years
Berger [40]	Bighorn sheep	≥ 100	-	70 years

Table S2 Biological data of thirty-six fish were used to estimate the parameters inputted in VORTEX model. All data were sourced from Wang et al. (2017).

Species	Life span (years)	Female age at maturity	Male age at maturity	Sex	L_∞ (cm)	t_0	k	Generation time (year)	Fecudity (egg)	Number of offspring per female	Instantaneous natural mortality rate at age 0-1 (from egg to age 1)
Odontobutis obscura	4	1	1	♀	15.29	0.1596	0.4072	1.5	491	1.69	0.997665
				♂	16.71	0.1095	0.3736				
Pseudobrama simoni	5	1	1	♀	19.98	-1.2118	0.1692	1.7	6681	1.23	0.999913
				♂	17.99	-1.3982	0.1766				

Ancherythroculter kurematsui	4	1	1		21.74	-0.7570	0.2867	1.7	2583	1.36	0.999521
Pelteobagrus fulvidraco	5	1	1	♀	17.90	-2.3300	0.1500	1.8	1173	1.15	0.999440
				♂	23.10	-1.2100	0.2200				
Sarcocheilichthys nigripinnis	4	1	1		17.23	-3.5300	0.1400	2.0	362	1.10	0.998020
Xenophysogobio boulengeri	5	1	1		19.55	-1.5100	0.1611	2.0	4547	1.19	0.999737
Botia superciliaris	5	2	2		16.70	-1.6209	0.1194	2.7	1907	1.84	0.999504
Glyptothorax fukiensis	5	1	1		14.02	-0.0696	0.2541	2.8	363	1.44	0.992558

Ancherythroculter nigrocauda	5	2	1	♀	28.44	-0.5900	0.2200	2.9	19962	2.42	0.999920
				♂	30.92	-0.2500	0.2500				
Pseudogobio vaillanti	6	2	2		23.80	-0.8050	0.2580	3.0	8535	2.35	0.999815
Paracanthobrama guichenoti	6	2	2		24.24	-1.4470	0.2643	3.0	9489	2.06	0.999828
Pseudobagrus truncatus	6	2	2	♀	44.37	-0.7082	0.1360	3.0	1488	2.16	0.999231
				♂	35.76	-1.2164	0.1524				
Pelteobagrus vachelli	8	2	2	♀	41.02	-0.7230	0.1638	3.0	4948	2.20	0.999731
				♂	53.51	-0.5279	0.1371				
Sinibrama macrops	6	2	1		24.04	-2.8540	0.1471	3.1	13266	1.46	0.999927

Squalidus argentatus	6	1	1	♀	17.18	-1.2673	0.1820	3.2	6121	1.02	0.999675
				♂	11.51	-0.5764	0.4074				
Coreius heterodon	8	2	2		60.02	-0.6108	0.2325	3.3	20308	2.44	0.999850
Misgurnus anguillicaudatus	6	1	1		28.65	-0.9968	0.1590	3.3	5336	1.02	0.999801
Cultrichthys erythropterus	6	1	1		30.03	-0.8300	0.2589	3.3	19752	1.14	0.999917
Leptobotia rubrilabris	7	2	2		22.02	-0.0530	0.2320	3.3	2340	3.58	0.998193
Channa argus	8	2	2		81.03	-0.5690	0.1755	3.5	15427	2.15	0.999901
Megalobrama pellegrini	7	2	2		48.89	-0.5453	0.1648	3.5	48032	2.24	0.999962

Xenocypris microlepis	6	2	1	37.04	-0.8939	0.2539	3.6	27199	2.12	0.999943
Culter alburnus	8	2	2	100.38	-0.4760	0.1807	3.8	306793	2.31	0.999991
Rhinogobio cylindricus	7	2	2	38.94	-0.7390	0.1770	3.9	9882	2.17	0.999564
Hypophthalmichthys molitrix	7	3	3	105.00	-0.0316	0.2046	4.2	302219	6.09	0.999984
Saurogobio dabryi	7	2	2	42.08	-2.5339	0.1160	4.2	15366	1.07	0.999958
Garra pingi	8	3	3	32.30	-1.4655	0.1781	4.3	2683	2.54	0.999141
Cyprinus (Cyprinus) carpio	7	2	1	97.26	-0.7841	0.2073	4.5	543652	1.77	0.999997
Paracanthobrama	7	4	4	72.04	-0.7788	0.1096	5.7	21247	3.91	0.999836

guichenoti											
Schizopygopsis malacanthus chengi	10	4	2	♀	32.32	-4.0240	0.0870	6.0	1568	1.10	0.999636
				♂	23.20	1.0080	0.2130				
Ctenopharyngodon idellus	9	4	3		121.50	-0.1915	0.1859	6.3	656053	5.87	0.999993
Hypophthalmichthys nobilis	8	4	3		139.30	-0.2513	0.1680	6.3	368816	5.17	0.999988
Schizothorax sinensis	10	5	4		58.00	-0.3766	0.1627	6.9	3537	6.93	0.998544
Silurus meriordinalis	13	3	2	♀	139.59	-1.5276	0.1474	7.0	80224	1.50	0.999983
				♂	119.48	-0.8962	0.2034				

Mylopharyngodon piceus	11	4	3		148.00	-0.1478	0.1541	8.1	681119	4.53	0.999993
				♀	313.75	-0.8240	0.0820				
Acipenser sinensis	34	13	8					21.8	516943	12.54	0.999988
				♂	255.23	-0.8420	0.1050				

Table S3 Changes of minimum viable population (MVP) estimated with 10%, 20% and 30% of S.D., respectively

Species	10%	20%	30%
Reproduction rate S.D.			
Hypophthalmichthys nobilis	320	333	335
Acipenser sinensis	180	180	181
Pelteobagrus vachelli	110	111	110
Culter alburnus	54	54	54
Pseudobrama simoni	42	46	47
Mortality rate S.D.			
Hypophthalmichthys nobilis	320	-	-
Acipenser sinensis	180	200	268
Pelteobagrus vachelli	110	151	260
Culter alburnus	54	69	107
Pseudobrama simoni	42	47	49

Table S4 Estimated minimum viable population (MVP) size and population growth rate (r) under different carrying capacity

Species	Carrying capacity (individual)	Population growth rate (r)	Minimum viable population (MVP)
Pseudobrama simoni	500	0.188	42
	1000	0.188	42
	2000	0.188	42
	5000	0.188	42
	10000	0.188	42
Acipenser sinensis	500	0.009	180
	1000	0.009	180
	2000	0.009	180
	5000	0.009	180
	10000	0.009	180
